# Impact of interface roughness correlation on resonant tunnelling diode variation

**DOI:** 10.1038/s41598-025-07720-0

**Published:** 2025-07-23

**Authors:** Pranav Acharya, Naveen Kumar, Ankit Dixit, Jaehyun Lee, Vihar Georgiev

**Affiliations:** 1 James Watt School of Engineering, University of Glasgow, Glasgow, G12 8QQ UK; 2 School of Electrical and Electronics Engineering, Pusan National University, Busan, South Korea

**Keywords:** Non-equilibrium Green’s function (NEGF), Resonant tunnelling diode (RTD), Interface roughness (IR), Electrical and electronic engineering, Electronic devices, Computational nanotechnology

## Abstract

The Nano-Electronic Simulation Software (NESS) features an improved model of Interface Roughness (IR), accounting for correlation lengths in two perpendicular directions and allowing anisotropic roughness. IR in $\text {GaAs/Al}_{0.3}\text {Ga}_{0.7}\text {As}$ Resonant Tunnelling Diodes (RTDs) was investigated using both the previous and improved models, with 4 correlation lengths ($L_C$) ranging from 2.5 nm to 10 nm. For each correlation length, 25 RTD device structures with IR were randomly generated. Device variation was quantified as the standard deviation of the resonant peak current ($I_r$) and the corresponding bias voltage ($V_r$), both extracted from the non-linear RTD current-voltage (IV) characteristics. The improved model resulted in greater variation, increasing standard deviation from 6.2 mV and 9 nA to 24.2 mV and 34.7 nA for $L_C=2.5$ nm. Standard deviation also roughly doubled as $L_C$ increased from 2.5nm to 10nm, increasing from 6.2 mV and 9 nA to 11.7 mV and 18.8 nA for the previous IR model, and from 24.2 mV and 34.7 nA to 38.8 mV and 80.9 nA for the improved IR model. A further study of anisotropic correlation lengths resulted in variation of standard deviations. This paper hence shows the importance of simulating IR with two correlation lengths for future accurate RTD research.

## Introduction

Roughness has become a significant source of variability in semiconductor devices as they have scaled down to nanometre dimensions^1,2^. The impact of roughness is particularly evident in Resonant Tunnelling Diodes (RTDs)^3–6^, which are highly sensitive to variations in the Quantum Well (QW)^7,8^ and barriers^8,9^, caused by Interface Roughness (IR)^10,11^ along heterostructure interfaces.

RTDs are quantum tunnelling based heterostructure devices with a non-linear current-voltage (IV) characteristic, which have drawn significant interest and research for their potential in THz electronics^5,6,12^. The Negative Differential Region (NDR) of RTDs, where current decreases as bias voltage increases, can compensate for losses in a resonator within an oscillator circuit^5,6^. Moreover, the speed of tunnelling allows such oscillators to reach THz frequencies at room temperature^13,14^. This makes RTDs strong contenders to bridge the ‘THz gap’^12^ with robust room temperature sources and detectors, enabling future applications such as high speed wireless communications for 6G^15^ and airport security imaging^16^.

As noted above, interface roughness is a key factor influencing RTD device performance^10,11^. However, research on the impact of IR on RTDs, including experimental studies, remains limited ^11,17,18^, including a lack of experimental data^10^. Additionally, previous simulation studies have treated IR simply as an additional scattering term^11,17,18^. To address this gap, the Nano-Electronic Simulation Software (NESS)^19^, a modular TCAD software under-development by the University of Glasgow, is used to study the variability in RTDs by randomly generating device structures with different IR profiles^20,21^. Specifically, we have implemented the Non-Equilibrium Green’s Function (NEGF)^22,23^ and Poisson solver modules within NESS^19^ to self-consistently simulate RTD devices. Furthermore, in this study, we introduce a new implementation of roughness, which can be generated using two correlation lengths along a plane, allowing anisotropic roughness and providing a more accurate model for practical devices. RTDs serve as an ideal test case for verifying this methodology used in simulation structure generation, due to their relatively simple structure.

Following this “Introduction” Section and the “Methodology” Section and “Results and discussion” Section, which are both split into three subsections, before finishing with the “Conclusion” Section. The “Methodology” Section first describes our use of the Nano-Electronic Simulation Software (NESS)^19^ developed in Glasgow University in the first subsection. The following subsection describes the simulation parameters along with a baseline ‘smooth’ RTD, and the last subsection describes the implementation of IR. The “Results and discussion” Section starts by describing the baseline ‘smooth’ RTD in the first subsection, compares the improved and previous implementations of IR in the following subsection, and studies anisotropic IR in the last subsection.

## Methodology

### Nano-electronic simulation software

NESS^19^ used in this study is a modular TCAD software under development by the University of Glasgow. To investigate the electrical performance of RTDs, the NEGF^22,23^ and Poisson equations are solved self-consistently. The use of the NEGF solver implemented in NESS allows for the inclusion of the quantum characteristics of RTDs, such as quantum tunnelling, in the device simulations. We employed the coupled-mode space method^23^ and finite difference approximations^24^ for these calculations. The recursive Green’s function algorithm^23,25^ and the Dyson equation^22,23^ calculate the retarded Green’s function and corresponding self-energies in the active region device-contact interfaces. The retarded (and advanced) Green’s function contains information about the allowed electronic states and hence can be used to calculate the spectral function^23^, the trace of which is the density of states. The self-energies take into account interactions and perturbations in the device, such as interactions with the contacts or electron scattering^23^. The lesser/advanced Green’s functions which are calculated from the retarded (and advanced) Green’s function and self-energies contain information about particle statistics, and can consequently be used to calculate charge and current density^23^. The finite difference approximation discretises the effective mass Hamiltonian^19^, resulting in a tri-diagonal one-particle Hamiltonian *h*^26^.

We calculate the retarded $G^\text {R}$, advanced $G^\text {A}$, and lesser/greater Green’s function $\,{G}^{\lessgtr }$ with the following equations:

1 $$\begin{aligned} G^{R}(E)&= \big [(E+i\eta )\cdot \mathbb {I} - h -\Sigma ^{R}(E)\big ]^{-1} \end{aligned}$$

2 $$\begin{aligned} G^{A}(E)&= \big [G^{R}(E)\big ]^{\dagger } \end{aligned}$$

3 $$\begin{aligned} G^{\lessgtr }(E)&=G^{R}(x,E) \cdot \Sigma ^{\lessgtr }(E) \cdot G^{A}(E) \end{aligned}$$

Here, *E* is the energy of the particle and $\Sigma ^{R}$ ($\Sigma ^{\lessgtr }$) is the leads’ retarded (lesser/greater) self-energy, where $\Sigma = \Sigma _{{\textbf {lead}}} + \Sigma _{{\textbf {scattering}}}$. $\eta$ is an infinitesimal positive real number and $\mathbb {I}$ is the identity matrix.

The electron density $n({\textbf {r}},E)$ and current density $J_{l \rightarrow l+1}(E)$ between layers *l* and $l+1$ are calculated using the lesser Green’s function, as noted below:

4 $$\begin{aligned} n({\textbf {r}},E)&= \frac{2}{2\pi }\int {dE\Im \big ( {\text {Tr}[G^{<}(r,r',E)]} \big )} \end{aligned}$$

5 $$\begin{aligned} J_{l \rightarrow l+1}(E)&=\frac{-2e}{\hbar } \int {\frac{dE}{2\pi } \times 2\Re \big (\text {Tr}[h_{l,l+1} G^{<}_{l+1,l}(E)]\big )} \end{aligned}$$

Here *e* is electron charge and $h_{l,l+1}$ $\big (G^{<}_{l+1,l}\big )$ are the matrix elements of the Hamiltonian (off-diagonal block of the lesser Green’s function) between the basis states on layer *l* ($l+1$) and $l+1$ (*l*). $\text {Tr}[...]$ is the trace of matrices and $\Re$ ($\Im$) is the real (imaginary) component.

**Fig. 1:**
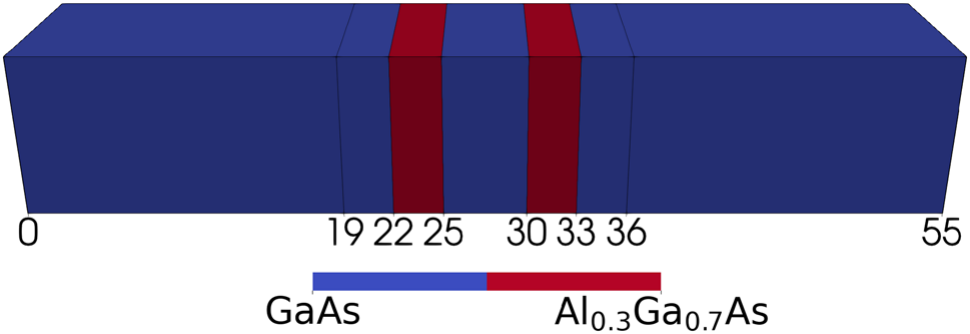
A schematic illustration of the baseline ‘smooth’ $\text {GaAs/Al}_{0.3}\text {Ga}_{0.7}\text {As}$ device with 3nm thick $\hbox {Al}_{0.3}\hbox {Ga}_{0.7}\hbox {As}$ barriers (shown in red) and a 5nm thick GaAs QW. The RTD cross-section is $10\,{\text{nm}} \times 10\,{\text{nm}}$ and the total length is 55nm. The 19nm thick source-drain regions are $2 \times 10^{{18}} \,{\text{cm}}^{{ - 3}}$ n-doped. The central 17nm region, including two 3nm buffers enclosing the barriers, is $1 \times 10^{{15}} \,{\text{cm}}^{{ - 3}}$ n-doped.

### Simulation parameters

The baseline ‘smooth’ RTD, as depicted in Fig. 1, is a GaAs nanowire with a length of 55 nm and a cross-section of $10\,{\text{nm}} \times 10\,{\text{nm}}$. The source-drain regions of this nanowire, each 19nm long, are n-doped to a concentration of $2\times 10^{18}\,{\text{cm}}^{-3}$ and enclose a central device region that is n-doped to $1\times 10^{15}\,{\text{cm}}^{-3}$. The central device region, located between 19 nm and 36 nm in Fig. 1), is composed of two 3nm thick spacer layers, one between 19 nm and 22 nm and the other between 33 nm and 36 nm, two 3 nm $\hbox {Al}_{0.3}\hbox {Ga}_{0.7}\hbox {As}$ barriers (coloured red in Fig. 1, located between 22 nm and 25 nm and between 30 nm and 33 nm), and a GaAs QW located between 25 nm and 30 nm. This high source-drain n-doping concentration is used to achieve a high current density^27^ and reduce the resonant peak voltage $V_r$ ^8^. The spacers are used to protect the central region from dopants diffusing into the central region^27,28^, which is important because dopant fluctuations lead to large effects in this central region^29^. The devices generated and simulated use a 0.2nm isotropic spatial mesh. The energies simulated range from a minimum energy of $E_{\text {min}} = 0.3$ eV below the drain contact, to a maximum energy of $E_{\text {max}} = 0.5$ eV above the Fermi level of the source contact. The resolution of the energy mesh is 1 meV and the device temperature is 300 K. For efficient statistical simulation, the ballistic approximation is used.

### Interface roughness generation

**Fig. 2:**
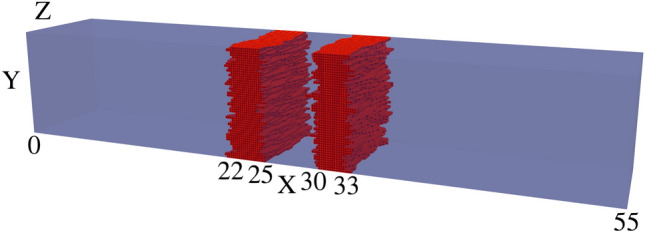
Visualisation of device 3 generated with an IR of correlation length $L_C=2.5$ nm, one of 25 such RTD devices. The rough $\hbox {Al}_{0.3}\hbox {Ga}_{0.7}\hbox {As}$ barriers (shown in red) are embedded within a GaAs (transparent blue) nanowire body.

**Fig. 3:**
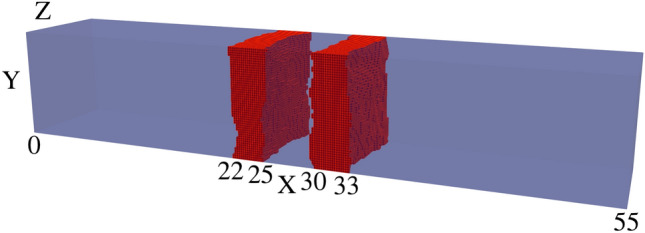
Visualisation of device 15 generated with ‘improved’ IR of isotropic correlation lengths $L^{Y}_{C}=L^{Z}_{C}=2.5$ nm, one of 25 such RTD devices. The rough $\text {Al}_{0.3}\text {Ga}_{0.7}\text {As}$ barriers (shown in red) are embedded within a GaAs (transparent blue) nanowire body.

**Fig. 4:**
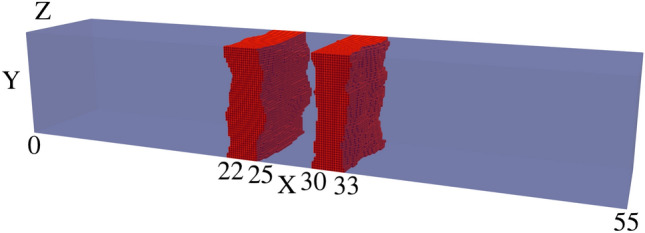
Visualisation of device 23 generated with ‘improved’ IR of anisotropic correlation lengths $L^{Y}_{C}=2.5$ nm and $L^{Z}_{C}=5$ nm, one of 25 such RTD devices. The rough $\text {Al}_{0.3}\text {Ga}_{0.7}\text {As}$ barriers (shown in red) are embedded within a GaAs (transparent blue) nanowire body.

**Fig. 5:**
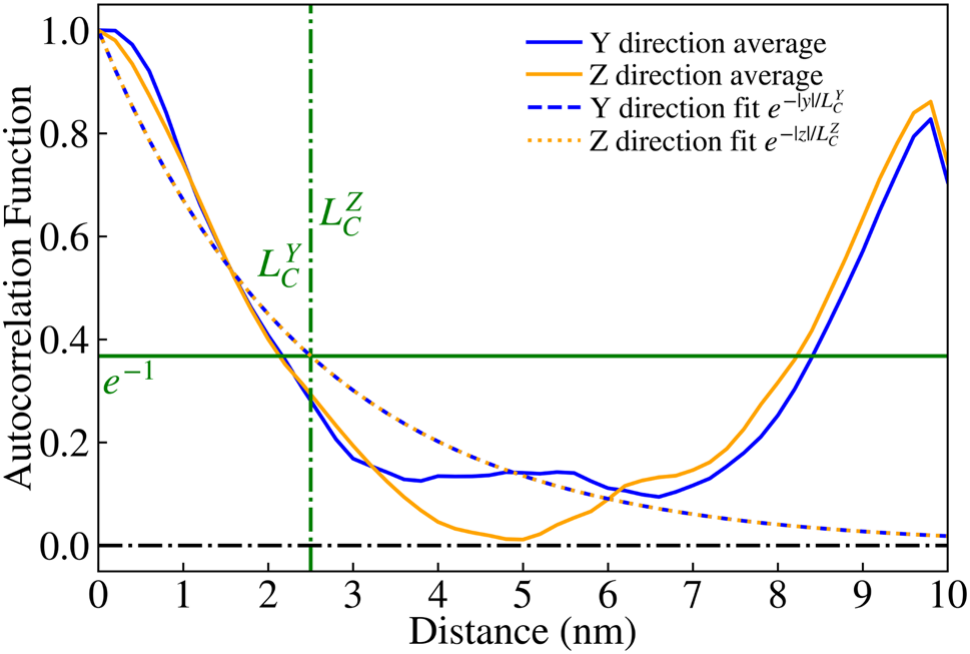
Average ACF for $\text {GaAs/Al}_{0.3}\text {Ga}_{0.7}\text {As}$ interfaces of 25 RTD devices generated in an ‘improved’ IR of isotropic correlation lengths $L^{Y}_{C}=L^{Z}_{C}=2.5$ nm, plotted as solid blue and yellow lines for the Y and Z directions respectively. The exponential ACF^30^ fits are given as dashed lines with the corresponding colours.

**Fig. 6:**
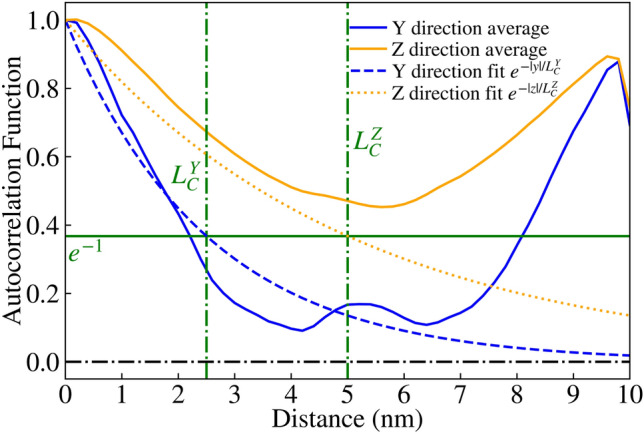
Average ACF for $\text {GaAs/Al}_{0.3}\text {Ga}_{0.7}\text {As}$ interfaces of 25 RTD devices generated in an ’improved’ IR of anisotropic correlation lengths $L^{Y}_{C}=2.5$ nm and $L^{Z}_{C}=5$ nm, plotted as solid blue and yellow lines for the Y and Z directions respectively. The exponential ACF^30^ fits are given as dashed lines with the corresponding colours.

NESS^20^ is capable of generating roughness by convolving a list of randomly generated numbers with either a Gaussian or exponential kernel, resulting in the corresponding Autocorrelation Function (ACF) with a specified correlation length $L_C$, as reported in literature^30^. This roughness is applied to a $\text {GaAs/Al}_{0.3}\text {Ga}_{0.7}\text {As}$ interface, and repeated for each such interface for a given device, resulting in devices with rough $\hbox {Al}_{0.3}\hbox {Ga}_{0.7}\hbox {As}$ barriers as seen in Figs. 2, 3 and 4. It is noteworthy that for the generated interfaces in this study, the variations are renormalised so that the standard deviation of the roughness asperity, $\Delta _{RMS}=0.3\,{\text{nm}}$, is close to the monolayer thickness for GaAs and $\hbox {Al}_{0.3}\hbox {Ga}_{0.7}\hbox {As/GaAs}$. Here, $\Delta _{RMS}=0.3\,\text{nm}$ corresponds to the fitted experimental values for $\hbox {Al}_{0.3}\hbox {Ga}_{0.7}\hbox {As/GaAs}$ QWs^31,32^. The correlation model chosen for this study was the exponential correlation model, which has been used before in literature^33^, where the ACF follows $e^{-|x/L_{C}|}$ as seen in Figs. 5 and 6.

**Fig. 7:**
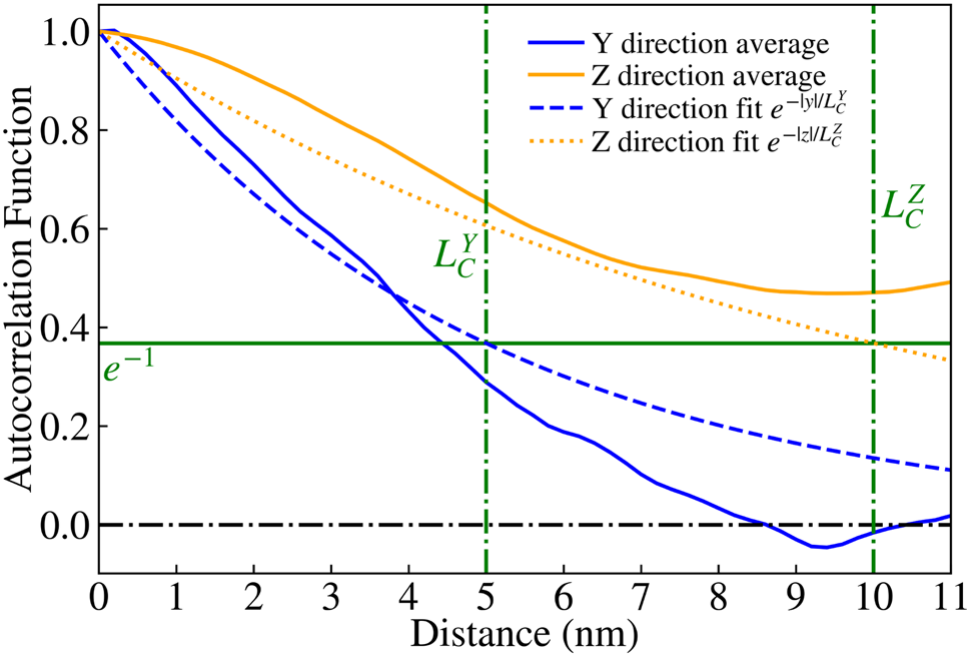
Average ACF for $\text {GaAs/Al}_{0.3}\text {Ga}_{0.7}\text {As}$ interfaces of 25 RTD devices generated in an ‘improved’ IR of anisotropic correlation lengths $L^{Y}_{C}=5$ nm and $L^{Z}_{C}=10$ nm and with a square cross-section of $20\,{\text{nm}} \times 20\,{\text{nm}}$, plotted as solid blue and yellow lines for the Y and Z directions respectively. The exponential ACF^30^ fits are given as dashed lines with the corresponding colours.

In the previous implementation of roughness, correlation was generated using a 1D randomly generated list, which was applied along one direction (the Y axis in this case) before incrementing along the other direction (the Z axis) and repeating the process. This resulted in devices like those shown in Fig. 2, with a correlation length of $L_{C}=2.5$ nm. We have expanded on this approach by including another correlation parameter, allowing for correlation along both axes with their own correlation lengths $L^{Y}_{C}$ and $L^{Z}_{C}$. The isotropic case of correlation lengths $L^{Y}_{C}=L^{Z}_{C}=2.5\,\text{nm}$ creates devices such as Fig. 3, and the anisotropic case of $L^{Y}_{C}=2.5\,\text{nm}$ and $L^{Z}_{C}=5\,\text{nm}$ creates devices such as Fig. 4. This correlation along both directions was achieved by taking the output from the correlated 1D list (with a kernel with a given $L^{Z}_{C}$ applied), effectively transposing so that the physical axes corresponding to each element ‘switches’, then applying correlation again (with a kernel for a given $L^{Y}_{C}$). The unusual artifact of increased ACF in comparison to the ACF fit (dashed lines) past a displacement of 5nm in Figs. 5 and 6 is due to the lack of data points for such a high displacement. Indeed, when the width of the square cross-section of the RTD was increased to 20nm, which is twice that of 10nm, the ACF was observed to follow the fit for up to 10nm, as illustrated in Fig. 7.

## Results and discussion

### Baseline ‘smooth’ RTD

**Fig. 8:**
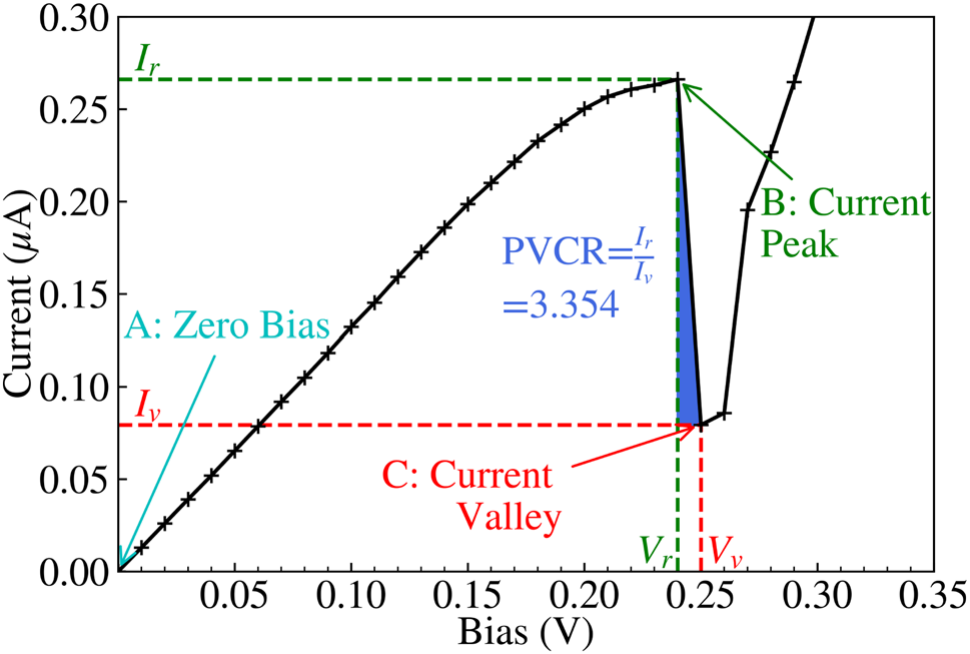
IV characteristic for the baseline RTD shown in Fig. 1. The resonant peak, or local maxima in current $I_r=0.2661$ μA at bias $V_r=0.24\,\text{V}$, and the valley, or local minima $I_v=0.0793$ μA at $V_v=0.25\,\text{V}$, are two key points for this nonlinear IV characteristic. These bound the NDR, and define the figure of merit PVCR (Peak-to-Valley Current Ratio) $I_r/I_v$. Figure 9a and b are measured for the resonant peak in this figure, and Fig. 9c and d are measured for the valley.

**Fig. 9:**
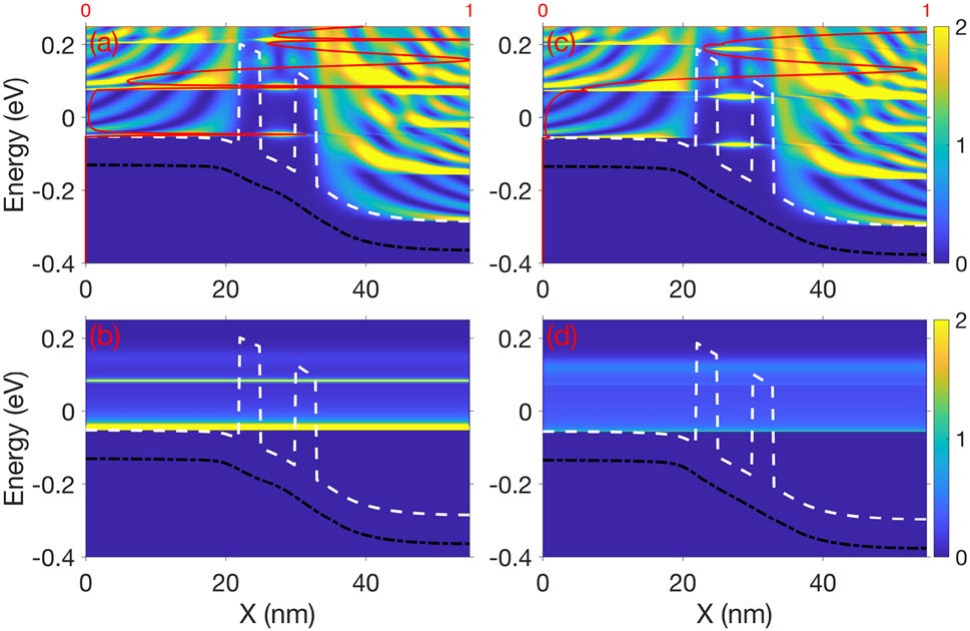
(**a**) depicts the LDOS and energy dependent transmission (a red vertical line) for the resonant peak at 0.24 V shown in Fig. 8, and (**b**) is the CS for the resonant peak. Similarly, (**c**) and (**d**) respectively represent the LDOS and CS for the valley at 0.25 V. The white dashed lines are the conductance band minima, and the black dashed lines are the average potential energy.

In this subsection, we explore the smooth baseline RTD depicted in Fig. 1, as a reference for comparing devices with IR. The IV characteristic displayed in Fig. 8 showcases the signature Negative Differential Resistance (NDR) region between the resonant peak $V_r=0.24\,\text{V}$ and the valley voltage $V_v=0.25\,\text{V}$, where current drops with increasing bias voltage. This drop in current is explained by the strong dependence of current on the resonant tunnelling of electrons through the QW previous-bound ground energy level for low bias, which depends on the alignment of the emitter-side Fermi level and said energy level. Fig. 9a and b are respectively the Local Density of States (LDOS) and Current Spectra (CS) of the RTD for the resonant peak along the x axis, and depict the resonant tunnelling phenomena. Fig. 9a also contains an energy dependent transmission red line, which peaks at the ground QW eigenvalue and corresponds to the band of CS shown in Fig. 9b. There is also a weaker CS band for the first excited QW eigenvalue in Fig. 9b. As bias increases to $V_v=0.25\,\text{V}$, the ground QW eigenvalue is brought below the emitter-side conduction band minima (white dashed line) shown in Fig. 9c. Because transverse momenta is conserved during tunnelling^34^, elastic tunnelling from the emitter Fermi level into the QW does not occur, suppressing transmission as seen in the energy dependent transmission, thereby leading to the reduced CS in Fig. 9d and the drop in current seen in Fig. 8. The space-charge effect^5^ causes the IV characteristic to have a greater negative resistance than otherwise, or a sharper drop in current from the resonant peak to the valley, leading to the distinctive ‘N’ shape seen in Fig. 8. The space-charge effect^5^ is the perturbation of the potential due to Coulomb repulsion from charge within the QW, which is proportional to the current flowing through it at any given time, which thus requires greater bias to overcome. This causes higher current regions in IV characteristics like Fig. 8 to perturb to greater bias.

**Fig. 10:**
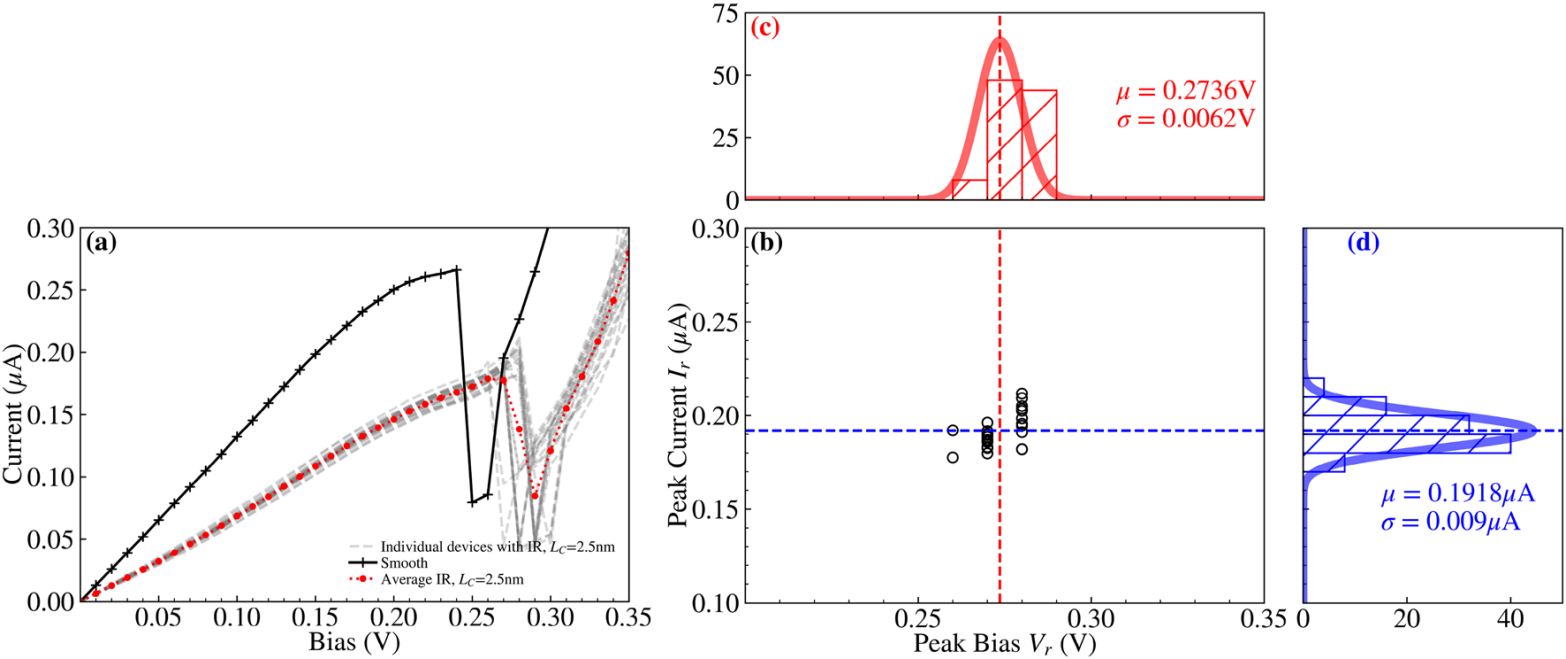
A composite figure visualising the IV characteristics of 25 RTDs generated with an IR of correlation length $L_C=2.5\,\text{nm}$. (**a**) depicts all the IV characteristics (grey dashed lines), with an average (red dotted line with dot markers) and a ‘smooth’ RTD (solid black line with plus markers) for comparison. (**b**) is a scatterplot of the resonant peak IV values taken from (**a**), and is bifurcated with dashed lines at the mean values of the resonant peak values, $V_r=0.2736\,\text{V}$ and $I_r=0.1918$ μA. (**c**) and (**d**) are accompanying histograms and fitted normal distributions for the resonant peak voltage $V_r$ and current $I_r$ distributions respectively.

**Fig. 11:**
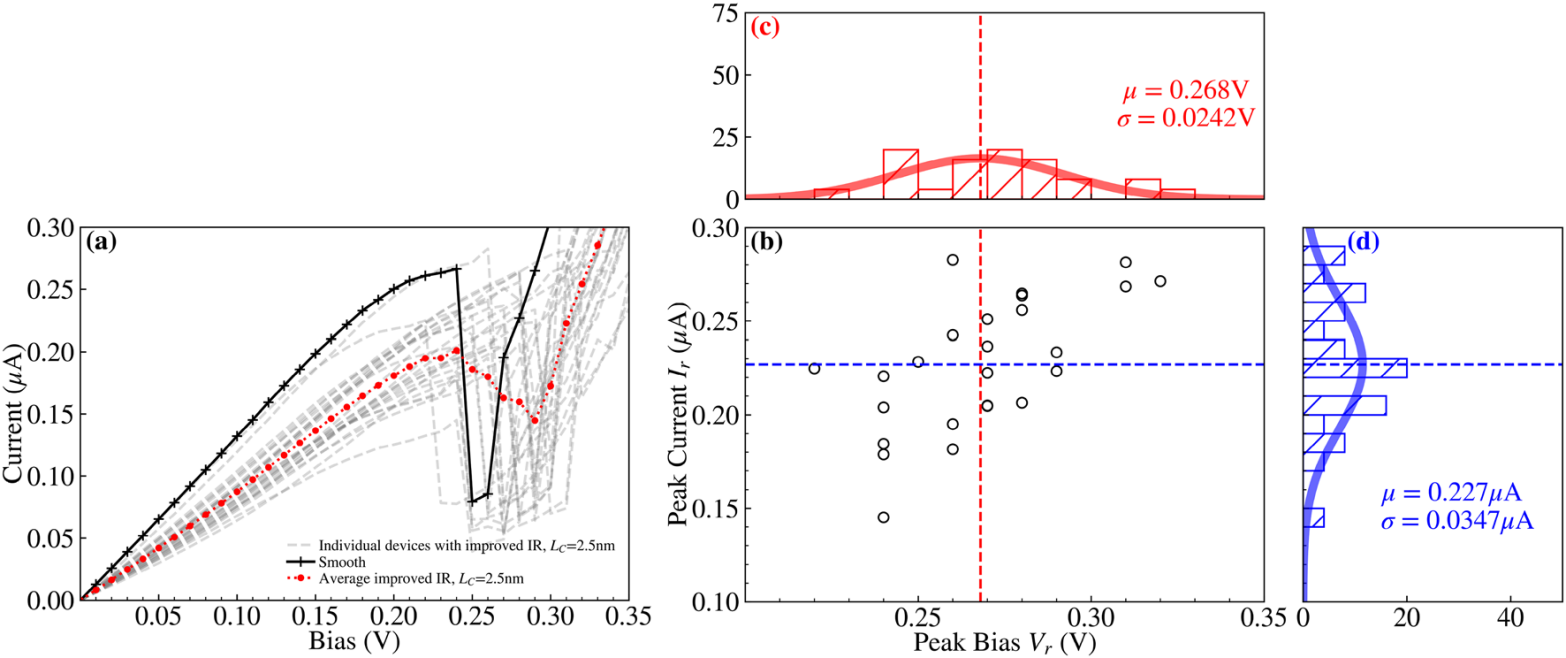
A composite figure visualising the IV characteristics of 25 RTDs generated with an ‘improved’ isotropic IR of correlation lengths $L^{Y}_{C}=L^{Z}_{C}=2.5\,\text{nm}$. (**a**) depicts all the IV characteristics (grey dashed lines), with an average (red dotted line with dot markers) and a ‘smooth’ RTD (solid black line with plus markers) for comparison. (**b**) is a scatterplot of the resonant peak IV values taken from (**a**), and is bifurcated with dashed lines at the mean values of the resonant peak values, $V_r=0.2680\,\text{V}$ and $I_r=0.2270$ μA. (**c**) and (**d**) are accompanying histograms and fitted normal distributions for the resonant peak voltage $V_r$ and current $I_r$ distributions respectively.

**Fig. 12:**
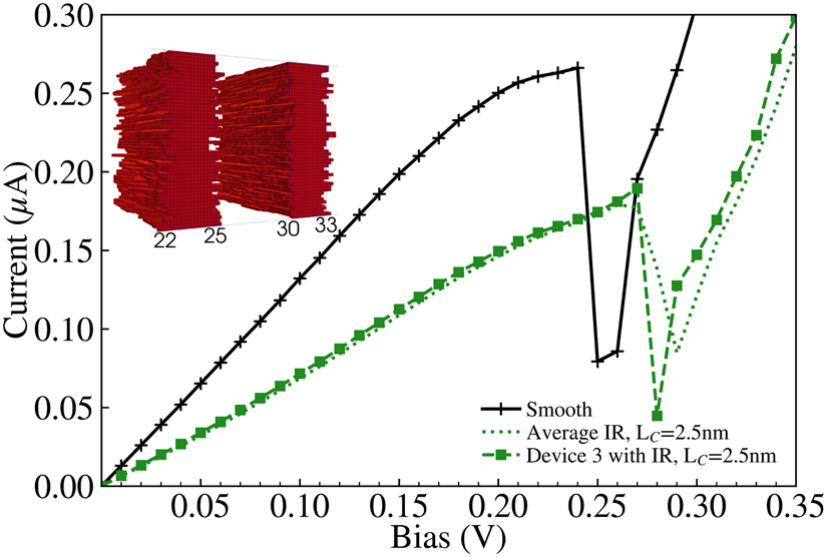
IV characteristics for device 3 (green dashed line with square markers) and the average (green dotted line) with IR of correlation length $L_C=2.5\,\text{nm}$, compared against the ‘smooth’ device IV characteristic (black solid line with plus markers). The inset contains the rough $\text {Al}_{0.3}\text {Ga}_{0.7}\text {As}$ barriers for device 3, which is shown in Fig. 2.

**Fig. 13:**
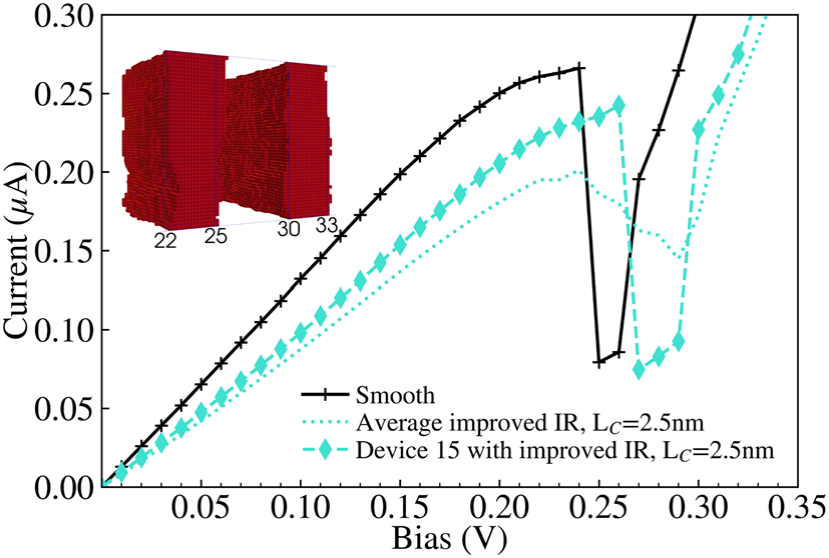
IV characteristics for device 15 (cyan dashed line with diamond markers) and the average (cyan dotted line) with ‘improved’ IR of correlation lengths $L^{Y}_{C}=L^{Z}_{C}=2.5\,\text{nm}$, compared against the ‘smooth’ device IV characteristic (black solid line with plus markers). The inset contains the rough $\text {Al}_{0.3}\text {Ga}_{0.7}\text {As}$ barriers for device 15, which is shown in Fig. 3.

### Improved isotropic interface roughness

**Table 1:** Mean $V_r$ and $I_r$ for the previous and ‘improved’ IR for different correlation lengths.

$L_C$ (nm)	IR		Improved IR	
	$V_{r}$ (V)	$I_{r}$ (μA)	$V_{r}$ (V)	$I_{r}$ (μA)
2.5	0.2736	0.1918	0.2680	0.2270
5.0	0.2764	0.1924	0.2716	0.2404
7.5	0.2788	0.1960	0.2640	0.2332
10.0	0.2748	0.1946	0.2536	0.2292

**Table 2:** Standard deviations of $V_r$ and $I_r$ for the previous and ‘improved’ IR for different correlation lengths.

$L_C$ (nm)	IR		Improved IR	
	$\sigma _{V_{r}}$ (mV)	$\sigma _{I_{r}}$ (nA)	$\sigma _{V_{r}}$ (mV)	$\sigma _{I_{r}}$ (nA)
2.5	6.2	9.0	24.2	34.7
5.0	6.9	10.2	28.7	47.6
7.5	11.4	17.2	35.9	68.8
10.0	11.7	18.8	38.8	80.9

We compare our previous implementation of IR with the ‘improved’ IR featuring isotropic correlation, while varying the correlation length from $L_C=2.5\,\text{nm}$ to $L_C=10\,\text{nm}$. Figures 10 and 11 illustrate the distribution of devices simulated with the previous and ‘improved’ IR models respectively, for $L_C=2.5\,\text{nm}$. Correspondingly, individual devices from these distributions are also presented in Figs. 12 and 13, and extracted values of the mean and standard deviation of resonant peak IV values are compared in Tables 1 and 2 respectively. Colourmaps depicting the resonant peak IV for the previous and ‘improved’ IR models are shown in the Supplementary Information file, as another way to visualise the data within Tables 1 and 2. Within the Supplementary Information file, Figures 1 and 2 visualise the mean and standard deviation of $I_r$ respectively, and Figures 3 and 4 similarly visualise the mean and standard deviation of $V_r$.

A comparison of Figs. 10 and 11 shows that the use of the ‘improved’ IR generation method results in greater variation in the IV characteristics and the corresponding resonant peak current and voltage. The standard deviation of the resonant peak voltage and current are nearly four times greater with the ‘improved’ IR, increasing from 6.2 mV and 9 nA to 24.2 mV and 34.7 nA as shown in Table 2. For both distributions, the average resonant peak perturbs to a greater bias and lesser current than the ‘smooth’ case of $I_r=0.2661$ μA at bias $V_r=0.24\,\text{V}$. As shown in Table 1, this perturbation in mean resonant peak values holds true for all the distributions simulated with IR. Additionally, for the ‘improved’ IR model, the mean $I_r$ experiences a lesser reduction compared to the previous IR model, with greater mean $I_r$ for all correlation lengths. Two specific device IV characteristics from these distributions of the previous and ‘improved’ IR are depicted respectively in Figs. 12 and 13, with the corresponding rough $\text {Al}_{0.3}\text {Ga}_{0.7}\text {As}$ barriers shown in their inset and the average IV characteristics (dotted lines) also plotted. Standard deviations of resonant peak values for both IR models roughly double in magnitude as correlation length increases from $L_C=2.5\,\text{nm}$ to $L_C=10\,\text{nm}$ as shown in Table 2, with an increase from 6.2 mV and 9 nA to 11.7 mV and 18.8 nA for the previous IR model, and an increase from 24.2 mV and 34.7 nA to 38.8 mV and 80.9 nA for the ‘improved’ IR model.

### Improved anisotropic interface roughness

**Table 3:** Standard deviations of $V_r$ (with units of mV) ‘improved’ anisotropic IR for different anisotropic correlation lengths.

$L^{Y}_C$ (nm)	$L^{Z}_{C}$ (nm)			
	2.5	5.0	7.5	10.0
2.5	24.2	29.1	25.3	26.2
5.0	25.1	28.7	25.2	32.9
7.5	32.3	25.9	35.9	30.7
10.0	26.1	32.7	31.3	38.8

**Table 4:** Standard deviations of $I_r$ (with units of nA) ‘improved’ anisotropic 2D IR for different anisotropic correlation lengths.

$L^{Y}_C$ (nm)	$L^{Z}_{C}$ (nm)			
	2.5	5.0	7.5	10.0
2.5	34.7	44.8	48.1	44.4
5.0	42.8	47.6	60.8	59.4
7.5	51.9	39.0	68.8	66.3
10.0	50.4	63.7	73.8	80.9

A new capacity of the ‘improved’ IR generation brought about by considering two correlation lengths is anisotropic roughness, which is demonstrated in Figs. 4 and 7. This is important because heterostructure interfaces often feature such anisotropic IR^35^. Therefore, as an extension of the previous subsection, we have measured and summarized standard deviations of resonant peak bias and current values for such distributions in Tables 3 and 4 respectively. The diagonal values of these tables are for ‘improved’ isotropic roughness, as noted in Table 2. From these tables, we found some variation in the standard deviations of resonant peak values, supporting the importance of including anisotropic roughness for simulations of devices with such roughness. Colourmaps of the mean and standard deviations of the resonant peak IV values are within the Supplementary Information file, to visualise the data within Tables 3 and 4, and to also depict the mean of the resonant peak IV values obtained. Within the Supplementary Information file, Figures 5 and 6 represent the mean and standard deviation of $I_r$ respectively for different anisotropic correlation lengths, and Figures 7 and 8 similarly represent the mean and standard deviation of $V_r$.

## Conclusion

We have presented an improved simulation of roughness using NESS by including two correlation lengths to generate roughness along a plane, and used this to investigate $\text {Al}_{0.3}\text {Ga}_{0.7}\text {As}/\hbox {GaAs}$ RTDs with IR along the $\text {Al}_{0.3}\text {Ga}_{0.7}\text {As}$ barriers. The improved IR simulation approach has resulted in RTDs exhibiting greater variation in the IV characteristics across distributions of 25 RTDs with different correlation lengths. This variation has been quantified numerically as the standard deviation of resonant peak voltage $V_r$ and current $I_r$. For a correlation length $L_C=2.5\,\text{nm}$, there is almost a four-fold increase in the standard deviations of resonant peak values, from 6.2 mV and 9 nA using the previous method to 24.2 mV and 34.7 nA with the improved IR method. Larger correlation lengths were found to increase the standard deviation for both the previous and the new method, with the standard deviation roughly doubling as $L_C$ increases from 2.5 nm to 10 nm. The improved IR generation method has also allowed us to measure standard deviations for anisotropic correlation, which exhibit variation with different correlation lengths. This increased variation and the ability to simulate anisotropic correlation lengths highlight the importance of this improvement to NESS, and suggests that future accurate simulations of device variation will require roughness with two correlation lengths. Future research directions includes studying in further detail the effects of anisotropic IR on RTDs and validating this IR model experimentally.

## Supplementary Information

Supplementary Information 1.

Supplementary Information 2.

## Data Availability

All data are available from the corresponding author upon request.

## Code availability

All codes are available from the corresponding author upon request.
