# Supplementary material for: Impact of interface roughness correlation on resonant tunnelling diode variation
Source: Sci Rep. 2025 Jul 23;15:26815. doi: 10.1038/s41598-025-07720-0 (PMC12287518; doi:10.1038/s41598-025-07720-0)
Supplement: Supplementary file 1 — Supplementary Information 1. [file 41598_2025_7720_MOESM1_ESM.pdf]

Supplementary Figures

Resonant peak IV (current-voltage) information for different distributions are represented as colourmaps here, including both standard deviations and the mean values of resonant peak current I_r and resonant peak voltage V_r.

Improved Isotropic Interface Roughness

The previous IR (Interface Roughness) and new IR models are compared in the colourmaps within this subsection.

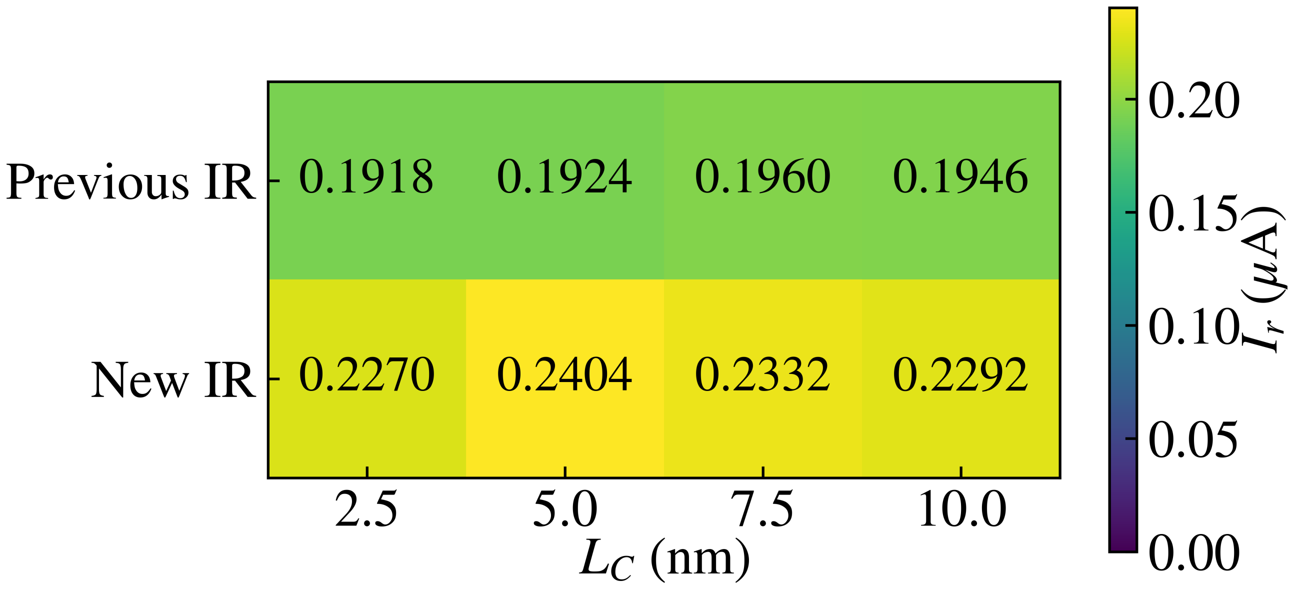

Figure 1. Mean of current peak I_r in microampere for different correlation lengths L_C for both the previous and new models of IR.

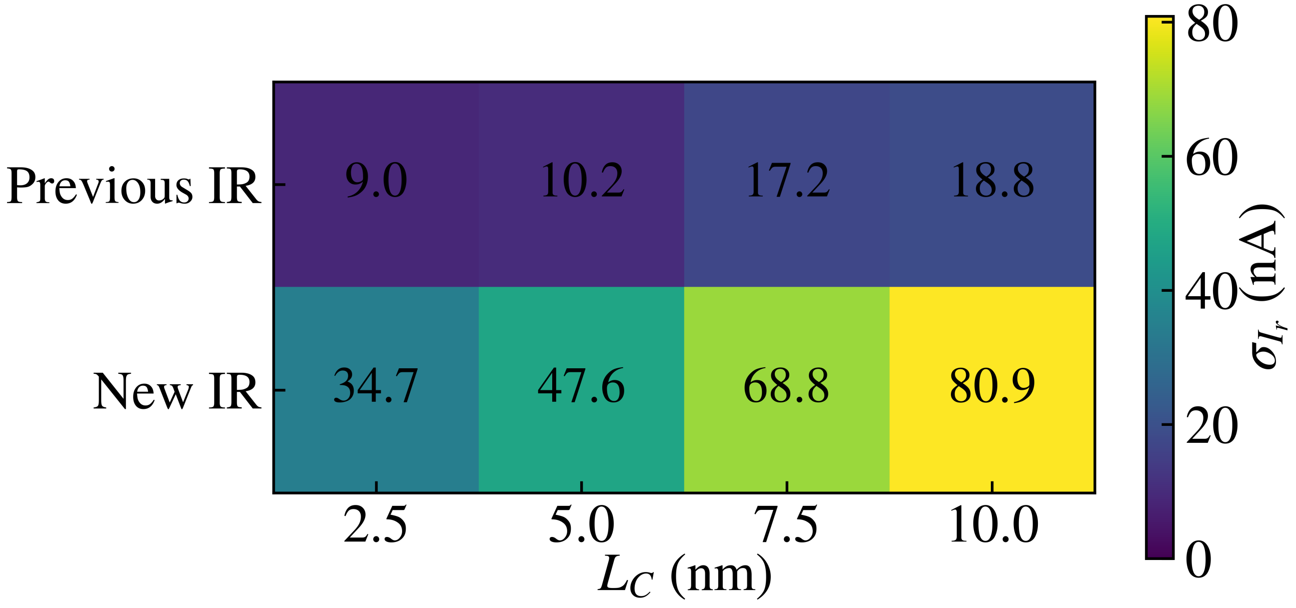

Figure 2. Standard deviation of current peak I_r in nanoampere for different correlation lengths L_C for both the previous and new models of IR.

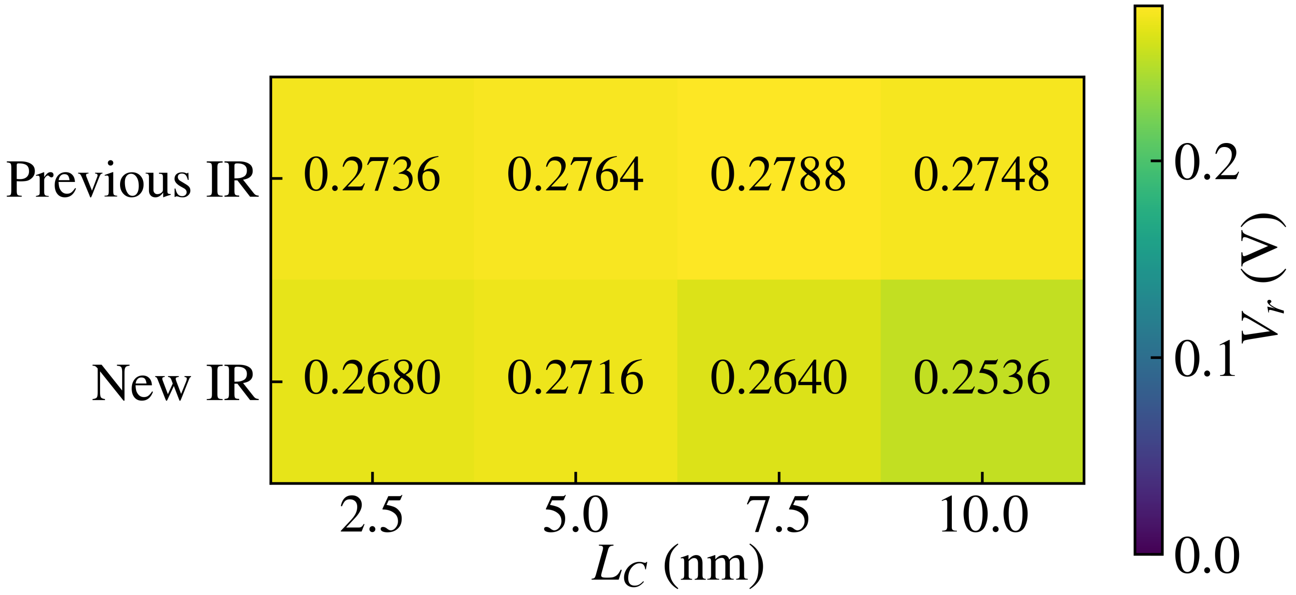

Figure 3. Mean of resonant voltage V_r in Volts for different correlation lengths L_C for both the previous and new models of IR.

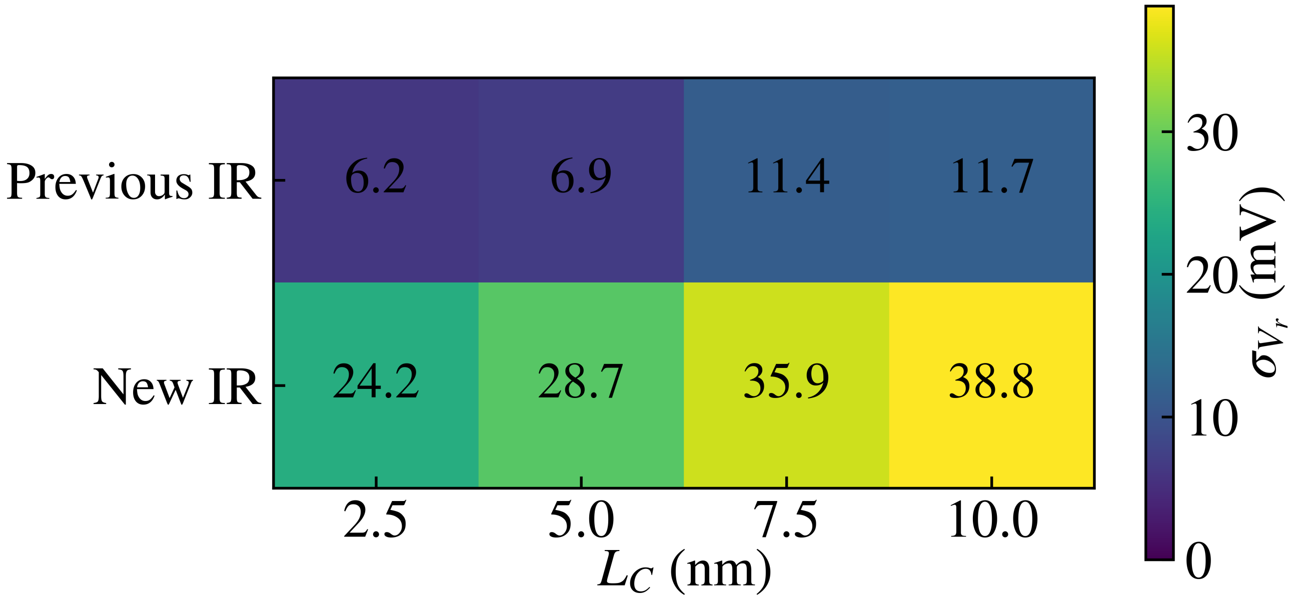

Figure 4. Standard deviation of resonant voltage V_r in millivolts for different correlation lengths L_C for both the previous and new models of IR.

Improved Anisotropic Interface Roughness

Mean and standard deviation of resonant peak IV for different anisotropic correlation lengths are displayed in the colourmaps in this subsection.

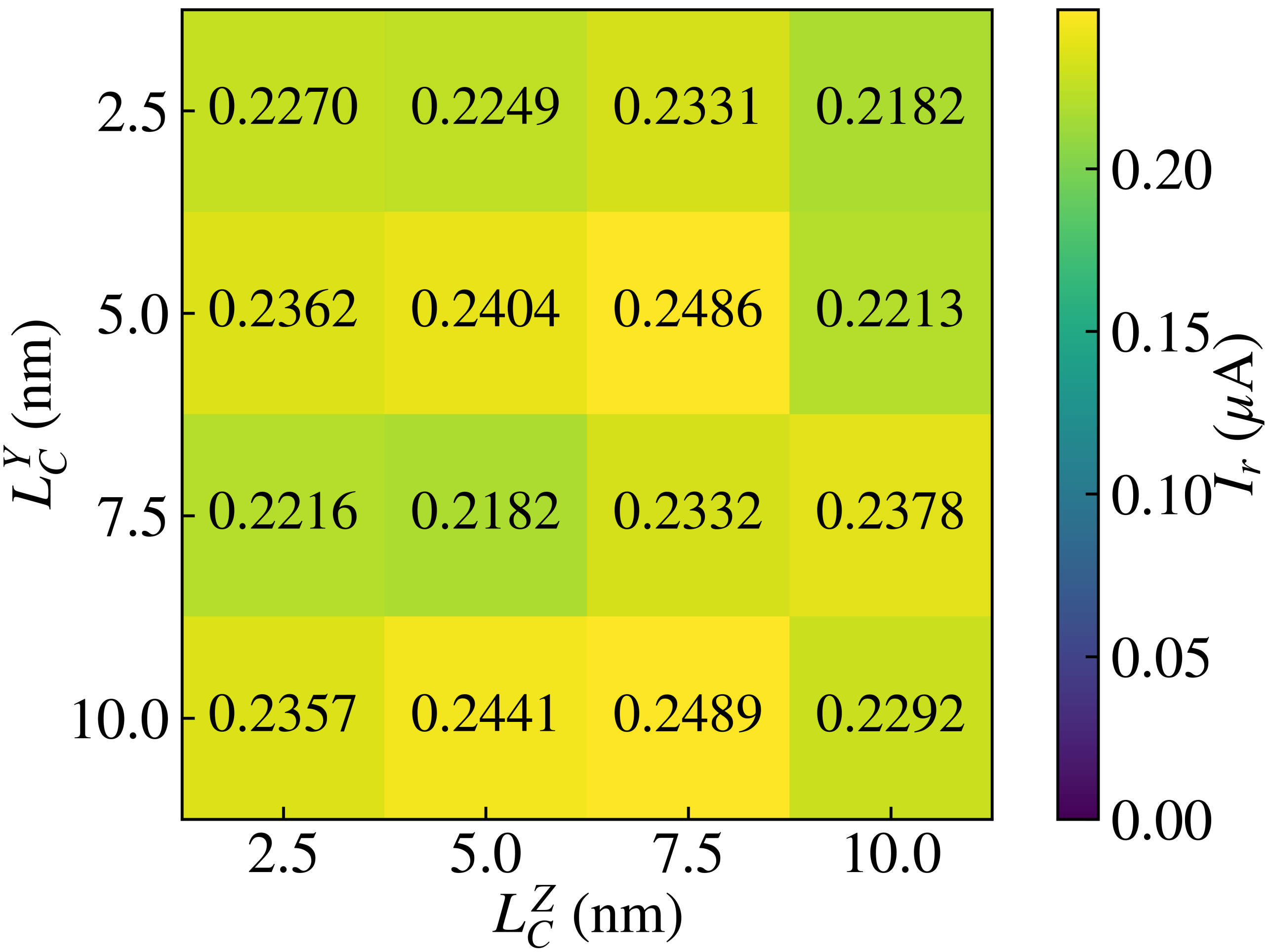

Figure 5. Mean of current peak I_r in microampere for different anisotropic correlation lengths L_C.

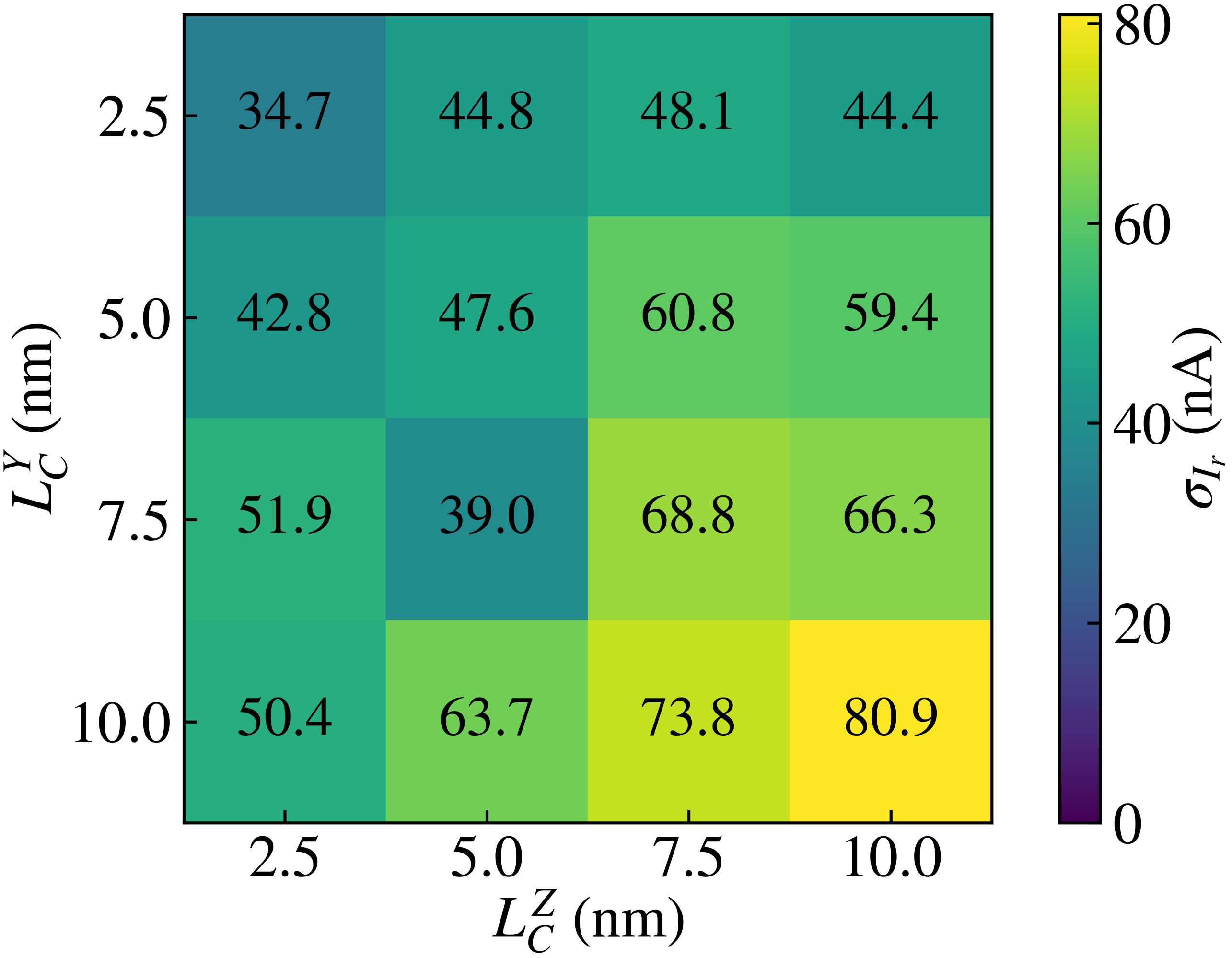

Figure 6. Standard deviation of current peak I_r in nanoampere for different anisotropic correlation lengths L_C.

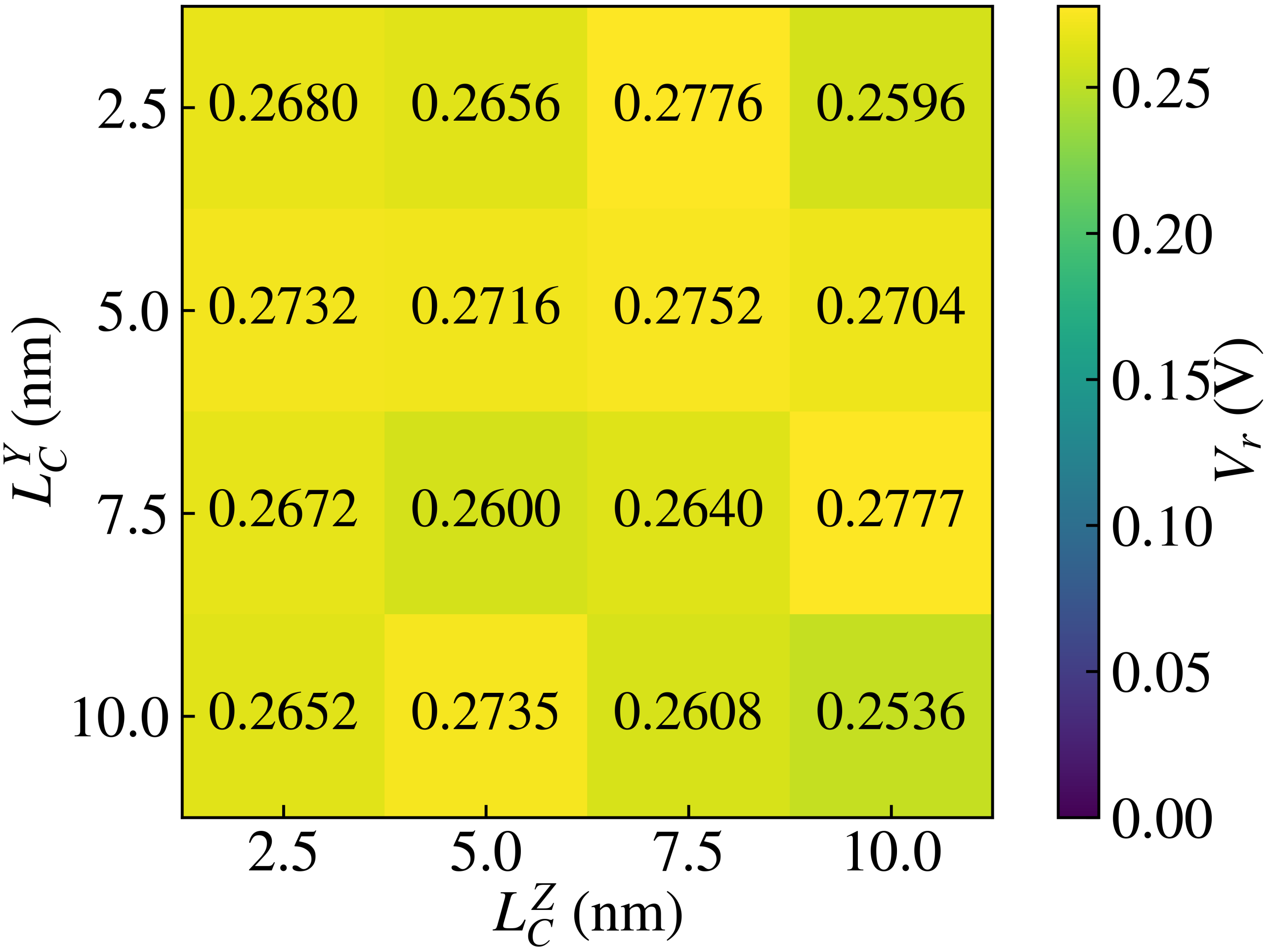

Figure 7. Mean of resonant voltage V_r in Volts for different anisotropic correlation lengths L_C.

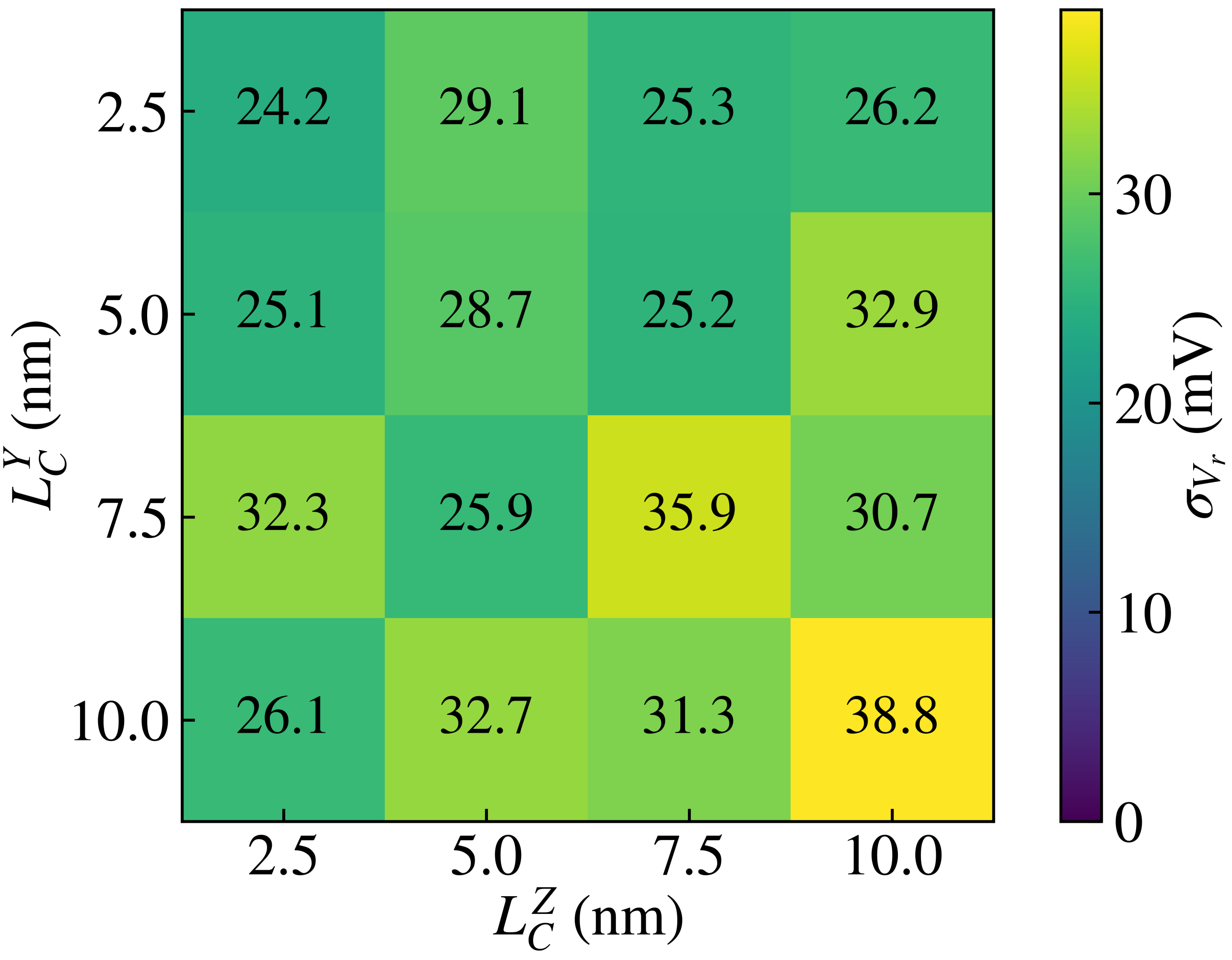

Figure 8. Standard deviation of resonant voltage V_r in millivolts for different anisotropic correlation lengths L_C.
