# Supplementary figures and images for: Impact of interface roughness correlation on resonant tunnelling diode variation
Source: Sci Rep. 2025 Jul 23;15:26815. doi: 10.1038/s41598-025-07720-0 (PMC12287518; doi:10.1038/s41598-025-07720-0)

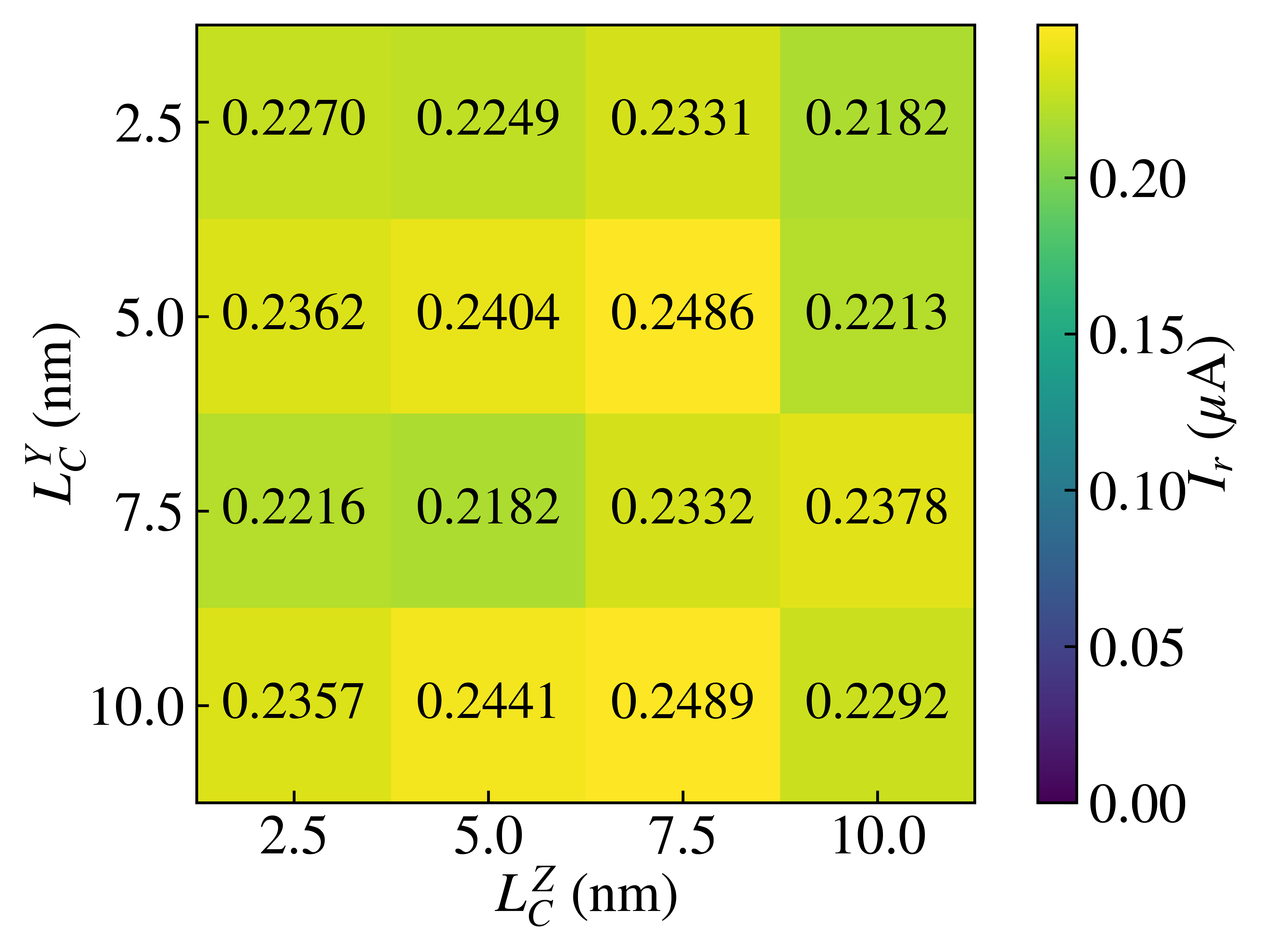

Supplement: Supplementary file 2 — Supplementary Information 2. [file 41598_2025_7720_MOESM2_ESM.zip › Supplementary_Information_Impact_of_IR_correlation_on_RTD_variation/Figures/grid_current_peak_mean.png]

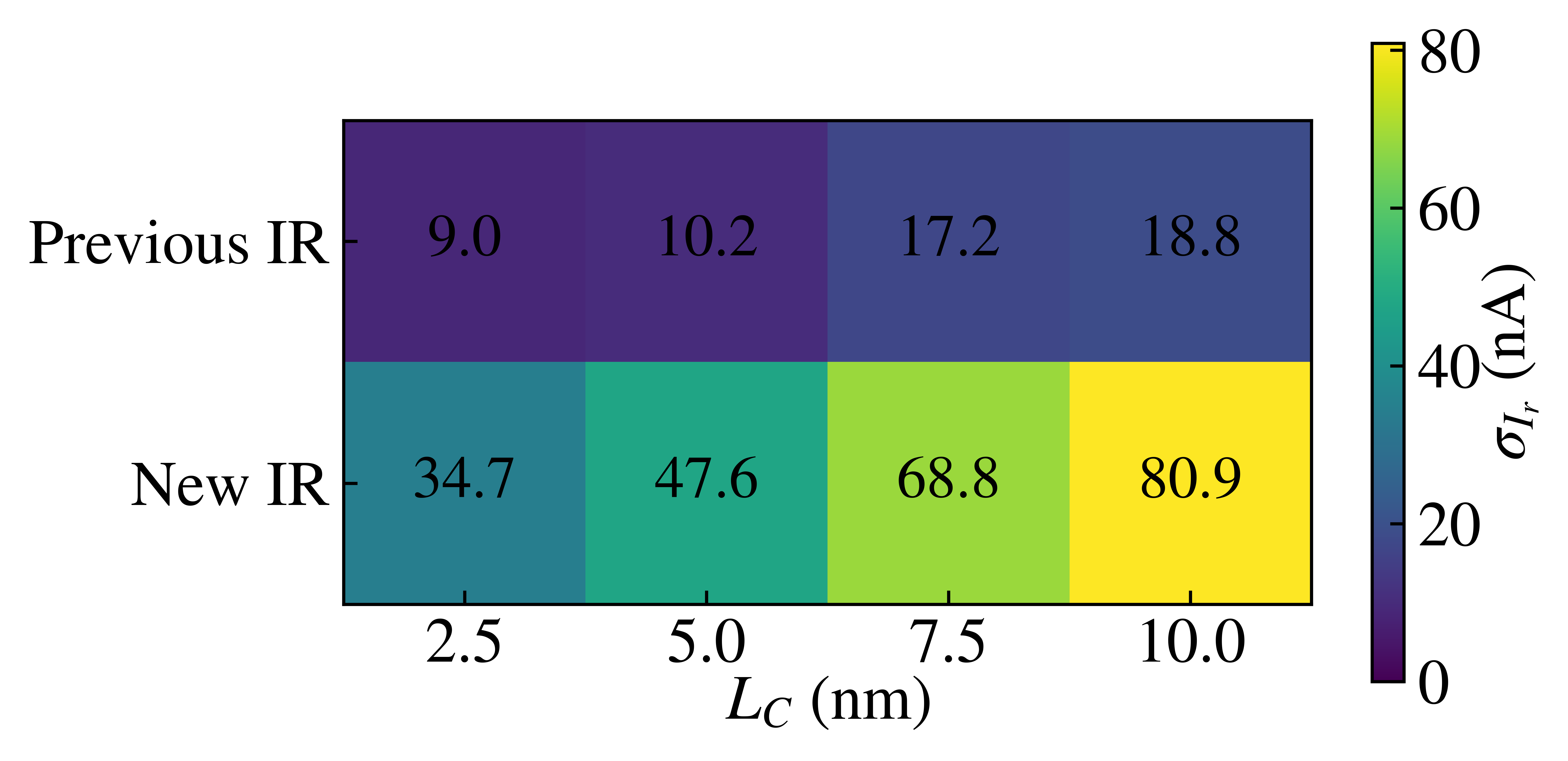

Supplement: Supplementary file 2 — Supplementary Information 2. [file 41598_2025_7720_MOESM2_ESM.zip › Supplementary_Information_Impact_of_IR_correlation_on_RTD_variation/Figures/comparison_previous_and_new_ir_current_peak_standard_deviation.png]

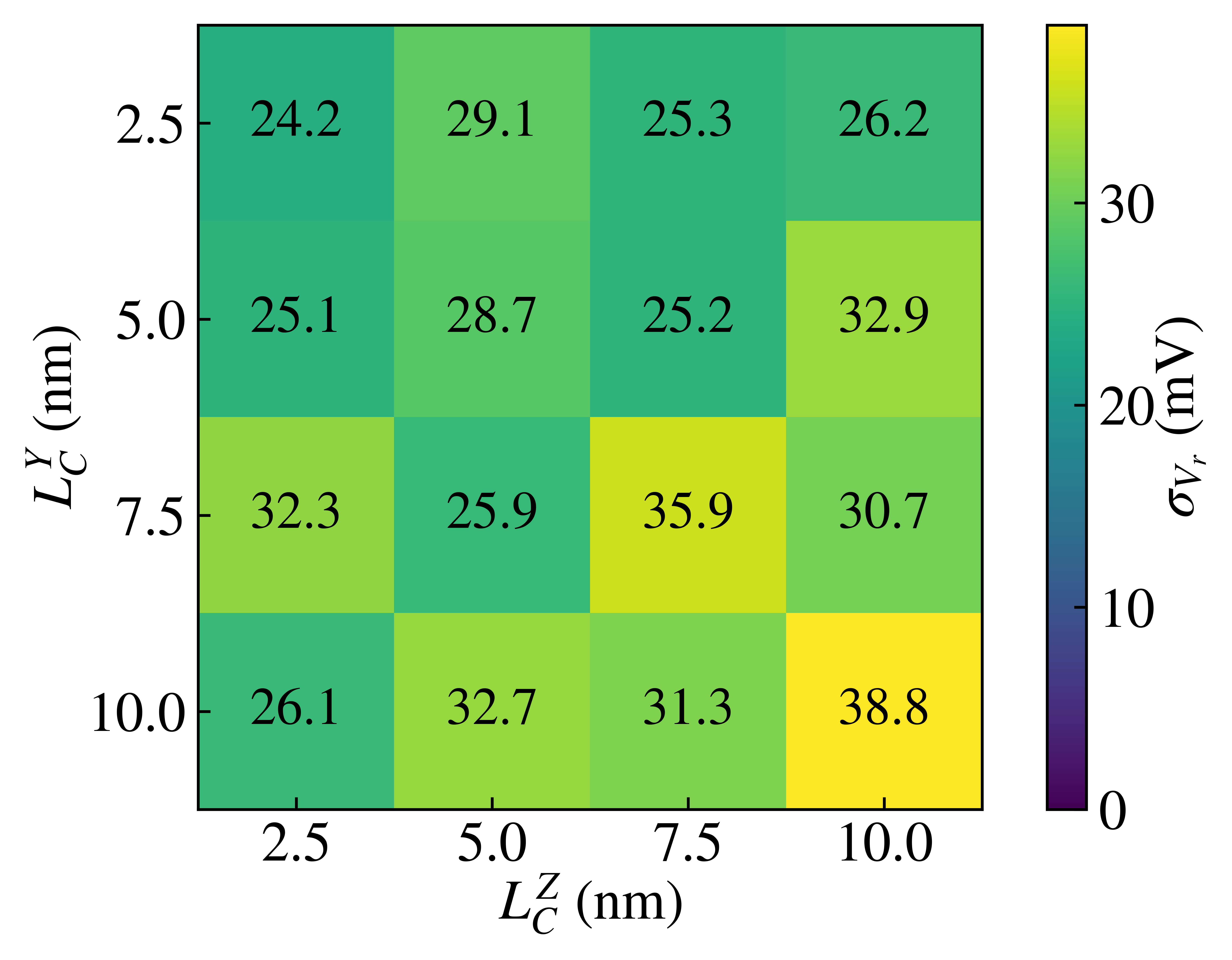

Supplement: Supplementary file 2 — Supplementary Information 2. [file 41598_2025_7720_MOESM2_ESM.zip › Supplementary_Information_Impact_of_IR_correlation_on_RTD_variation/Figures/grid_voltage_peak_standard_deviation.png]

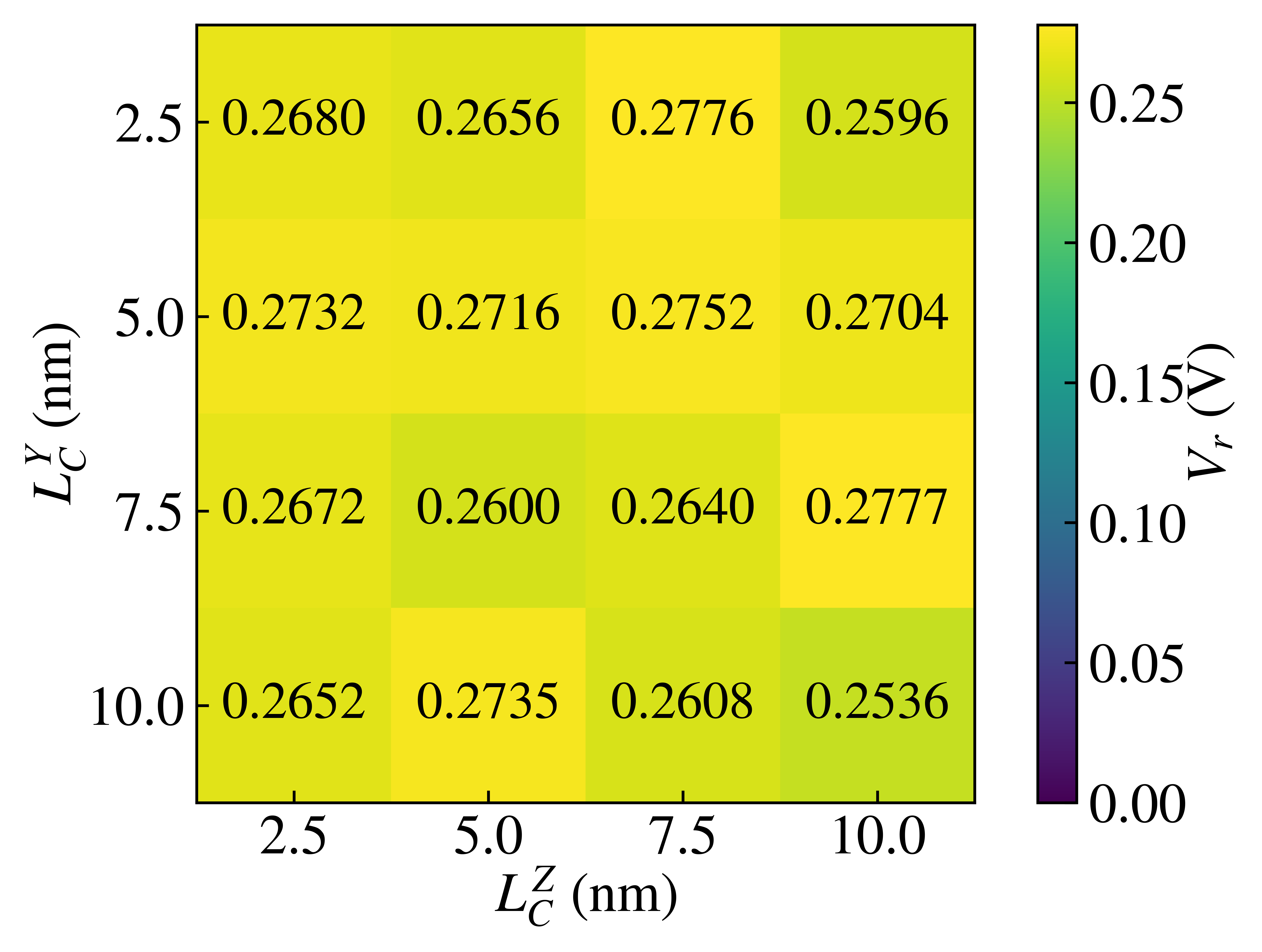

Supplement: Supplementary file 2 — Supplementary Information 2. [file 41598_2025_7720_MOESM2_ESM.zip › Supplementary_Information_Impact_of_IR_correlation_on_RTD_variation/Figures/grid_voltage_peak_mean.png]

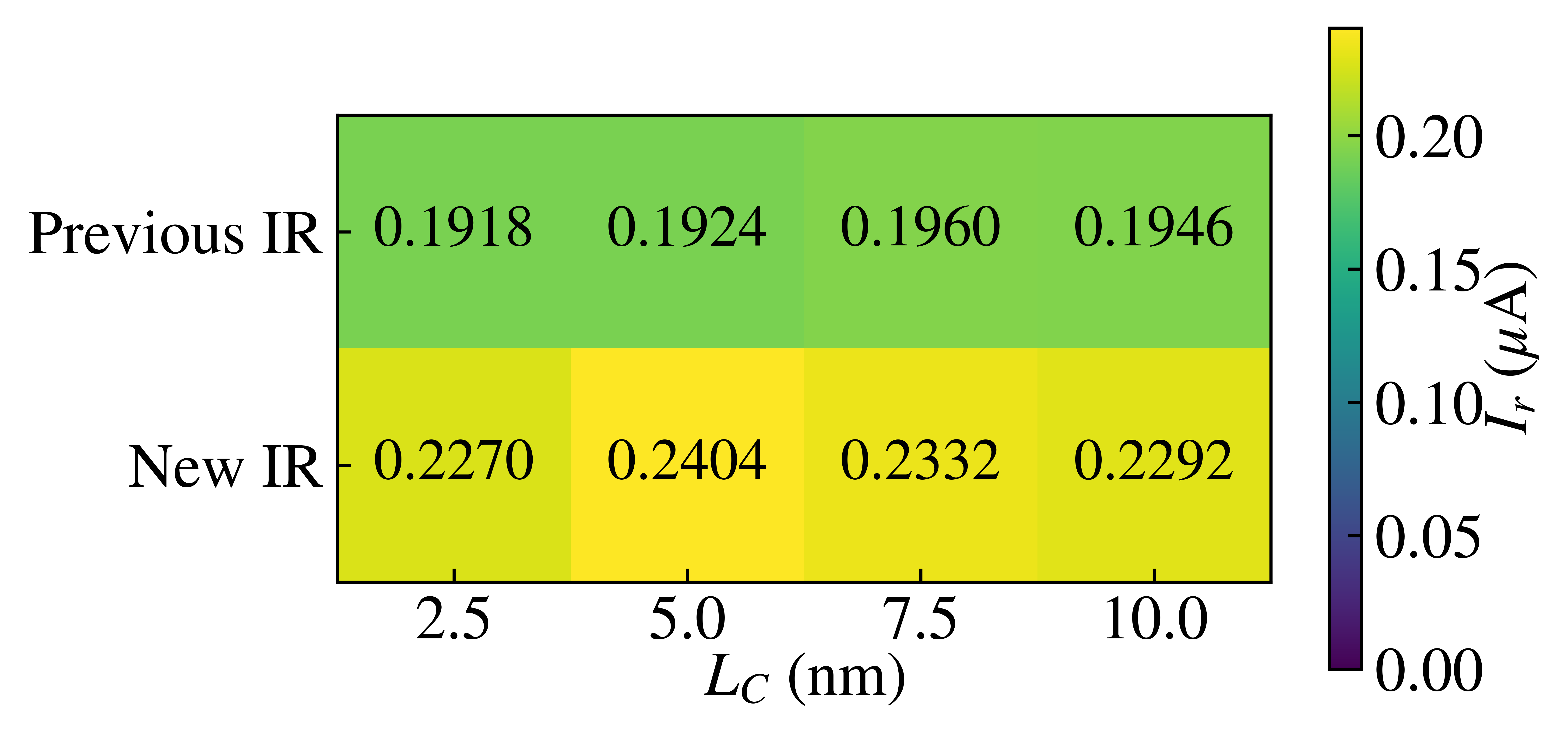

Supplement: Supplementary file 2 — Supplementary Information 2. [file 41598_2025_7720_MOESM2_ESM.zip › Supplementary_Information_Impact_of_IR_correlation_on_RTD_variation/Figures/comparison_previous_and_new_ir_current_peak_mean.png]

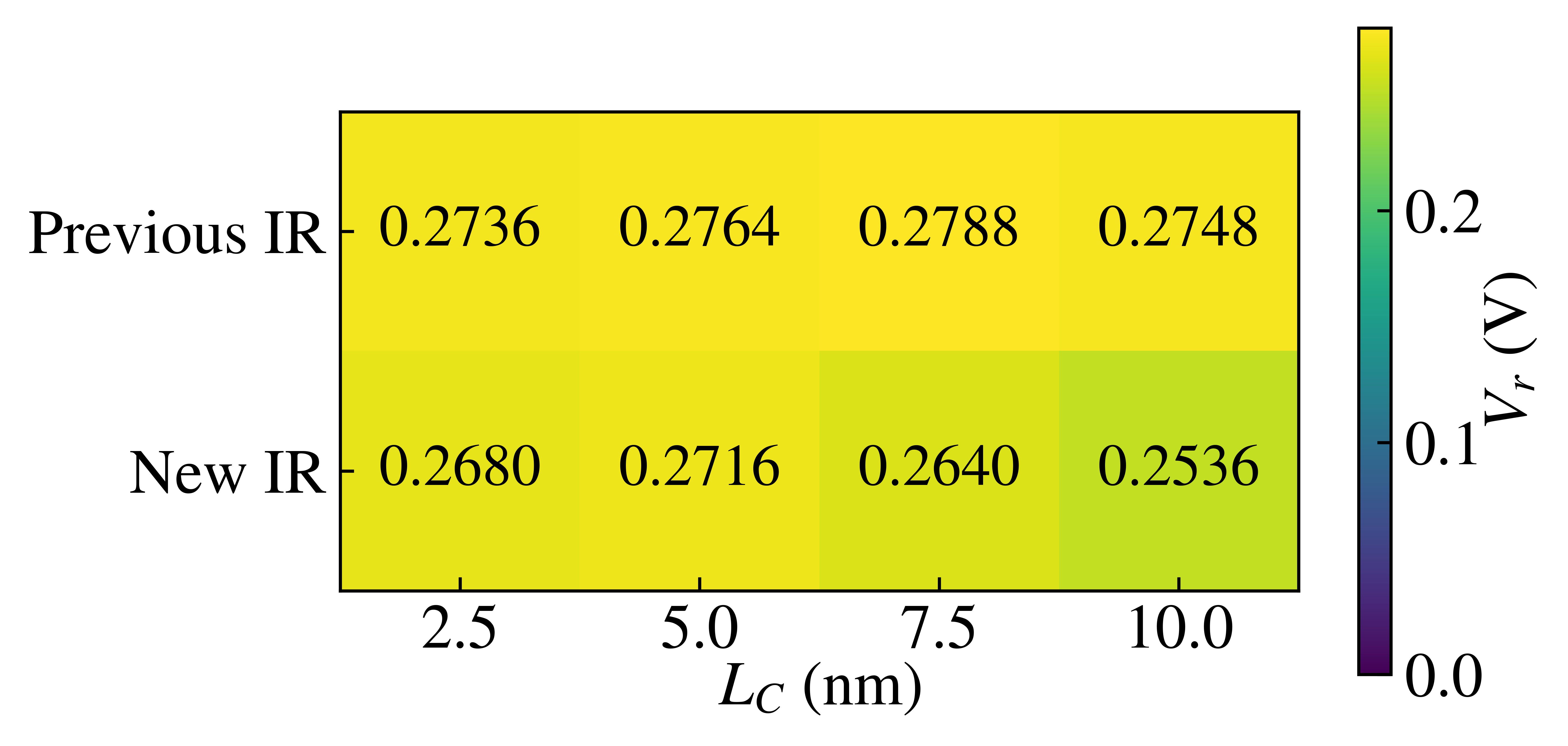

Supplement: Supplementary file 2 — Supplementary Information 2. [file 41598_2025_7720_MOESM2_ESM.zip › Supplementary_Information_Impact_of_IR_correlation_on_RTD_variation/Figures/comparison_previous_and_new_ir_voltage_peak_mean.png]

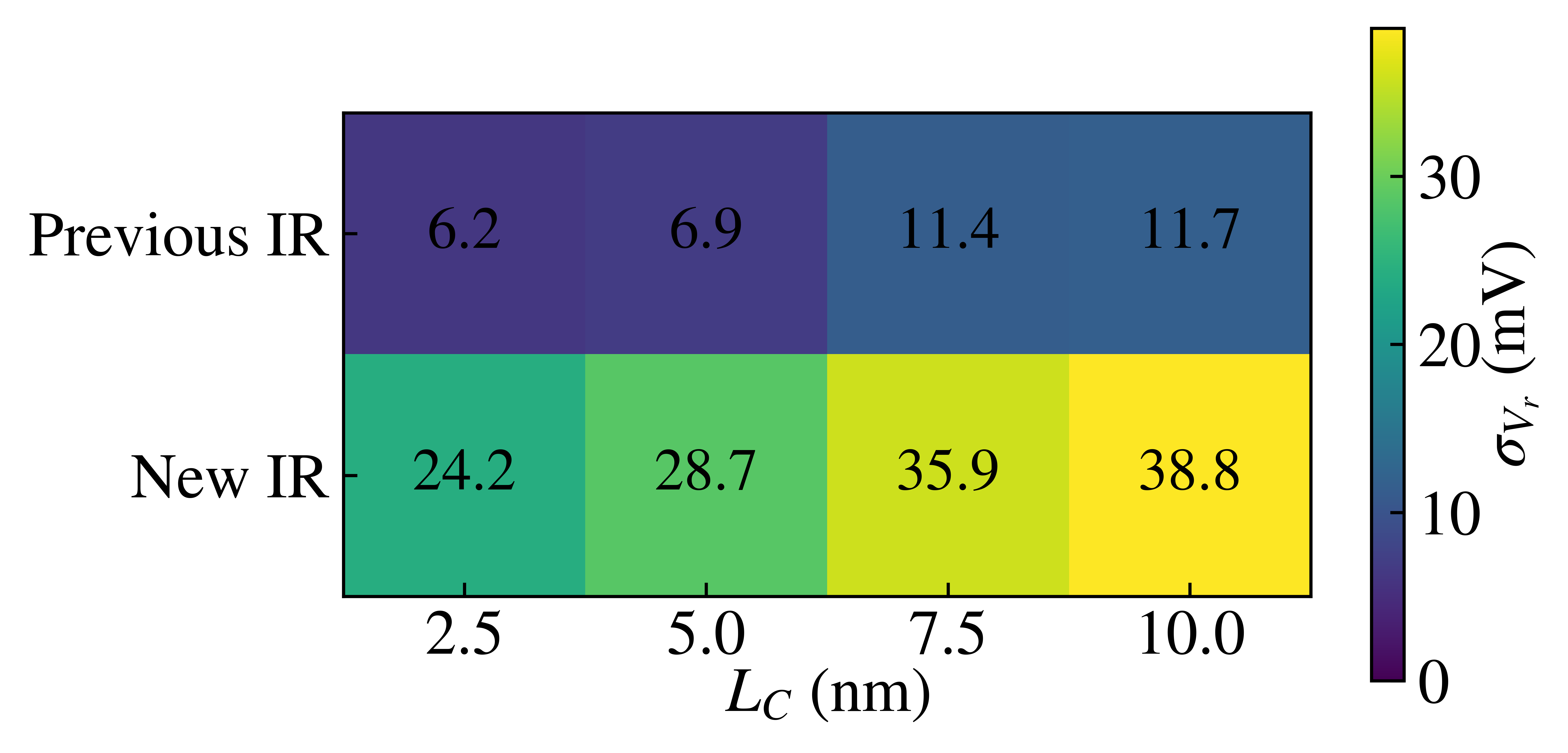

Supplement: Supplementary file 2 — Supplementary Information 2. [file 41598_2025_7720_MOESM2_ESM.zip › Supplementary_Information_Impact_of_IR_correlation_on_RTD_variation/Figures/comparison_previous_and_new_ir_voltage_peak_standard_deviation.png]

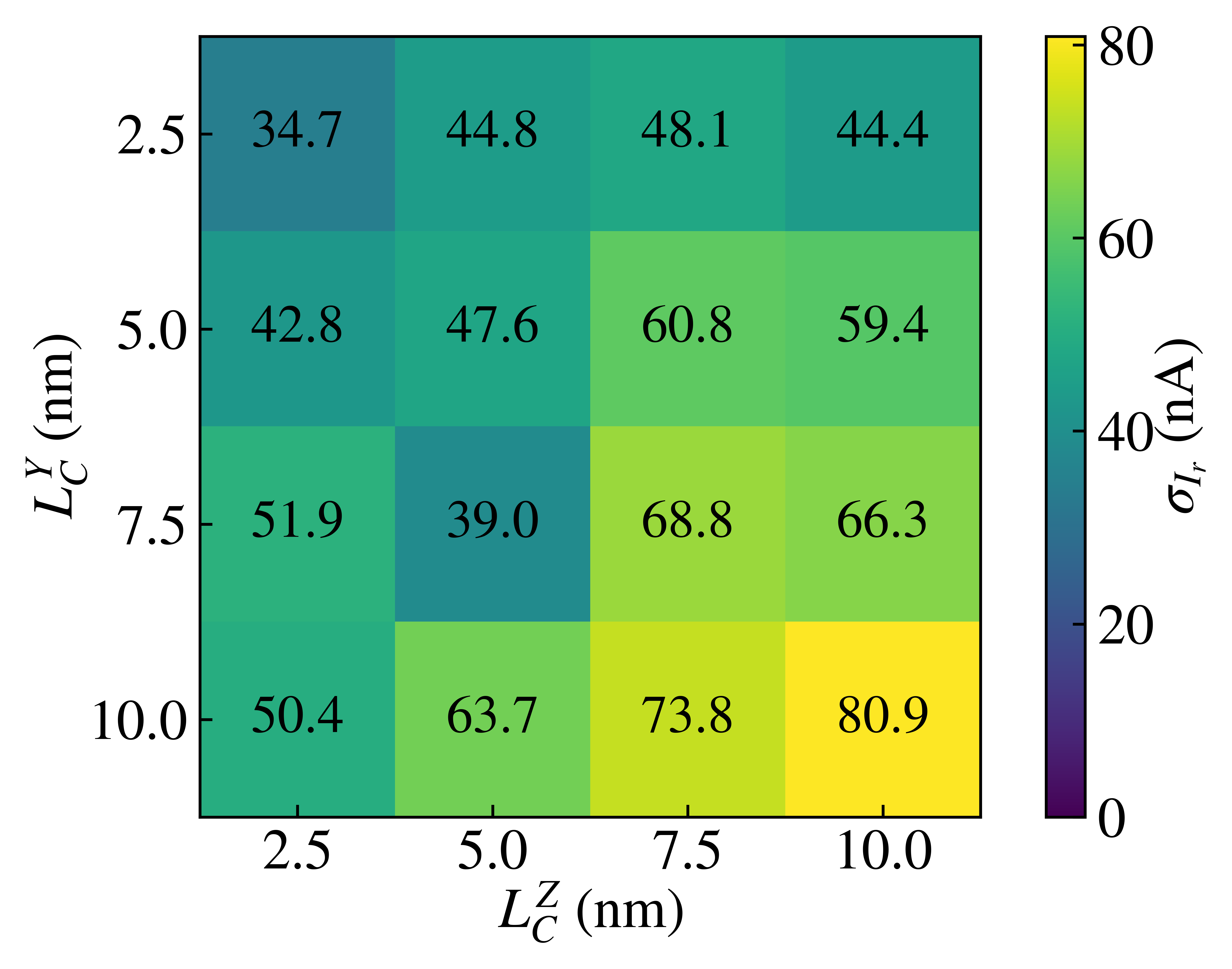

Supplement: Supplementary file 2 — Supplementary Information 2. [file 41598_2025_7720_MOESM2_ESM.zip › Supplementary_Information_Impact_of_IR_correlation_on_RTD_variation/Figures/grid_current_peak_standard_deviation.png]
